# Effects of Environment and Space on Species Turnover of Woody Plants across Multiple Forest Dynamic Plots in East Asia

**DOI:** 10.3389/fpls.2016.01533

**Published:** 2016-10-13

**Authors:** Yun Chen, Zhiliang Yuan, Peikun Li, Ruofan Cao, Hongru Jia, Yongzhong Ye

**Affiliations:** ^1^College of Forestry, Henan Agricultural UniversityZhengzhou, China; ^2^College of Life Sciences, Henan Agricultural UniversityZhengzhou, China; ^3^Educational Administration Department, Henan University of Finance and BankingZhengzhou, China

**Keywords:** climate, environmental, latitudinal gradient, neutral processes, growth forms

## Abstract

Species turnover is fundamental for understanding the mechanisms that influence large-scale species richness patterns. However, few studies have described and interpreted large-scale spatial variation in plant species turnover, and the causes of this variation remain elusive. In addition, the determinants of species turnover depend on the dispersal ability of growth forms. In this study, we explored the large-scale patterns of woody species turnover across the latitude gradient based on eight large stem-mapping plots (covering 184 ha forest) in East Asia. The patterns of woody species turnover increased significantly with increasing latitude differences in East Asia. For overall woody species, environment explained 36.30, 37.20, and 48.48% of the total variance in Jaccard’s (β_j_), Sorenson’s, (β_s_), and Simpson’s dissimilarity (β_sim_). Spatial factors explained 47.92, 48.39, and 41.38% of the total variance in β_j_, β_s_, and β_sim_, respectively. The effects of pure spatial and spatially structured environments were stronger than pure environmental effects for overall woody species. Our results support the hypothesis that the effect of neutral processes on woody species turnover is more important than the effect of the environment. Neutral processes explained more variation for turnover of tree species, and environmental factors explained more variation for the turnover of shrub species on a large scale. Therefore, trees and shrubs should be subjected to different protection strategies in future biodiversity conservation efforts.

## Introduction

Species turnover pattern (or beta diversity) is a basic pattern in biogeography and macroecology (Gaston, 2000), and it provides fundamental insights into mechanisms of community assembly, especially on a large scale (Anderson et al., 2011). However, species turnover patterns have received less attention than alpha diversity (Koleff et al., 2003), and most studies on species turnover have been conducted locally (Kraft et al., 2011; Wang et al., 2012a). On a large scale, recent studies have demonstrated that species spatial turnover is a key factor for both plants and animals (Kreft and Jetz, 2007; Qian and Ricklefs, 2008). Stuart et al. (2012) have found that the environmental dissimilarity coefficient of lizards and frogs increased with changing geographical distance and habitat; Qian et al. (2009) have shown that the similarity coefficients of North American mammals and African amphibians decrease with increasing spatial distance. However, the studies on the patterns of species turnover across latitudes are relatively few. Moreover, in the few studies about the patterns of species turnover across latitudes, a great majority is based on the space-filling species of range maps. The drawbacks of this method include coarse spatial resolutions; spatial heterogeneity among species composition has been smoothed out, and spatial autocorrelation has increased (Wang et al., 2012b).

The underlying mechanisms governing large-scale species turnover are ambiguous and poorly studied (De Cáceres et al., 2012) despite several and recent efforts to describe large-scale species turnover patterns of plant (De Cáceres et al., 2012; Tang et al., 2012a,b; Wang et al., 2012b; Xu et al., 2015). Several research studies have indicated that species coexistence is attributed to different environmental factors; species have different resources, time, and space to achieve coexistence (Jia et al., 2015; Escudero and Valladares, 2016). Alternatively, neutral processes state that species coexistence results from biogeographic barriers and low dispersal abilities (Hubbell, 2001; Jia et al., 2015). Moreover, results vary because of differences in the methods, including the spatial extents and the measures of species turnover. Therefore, the relative role of the environment and neutral processes in governing species turnover may vary (Liu et al., 2015; Valladares et al., 2015).

The dispersal ability of plant species is frequently predicted to influence species turnover, but only limited tests have been performed to confirm this; the results of these tests have been inconsistent (Qian, 2009; Elgar et al., 2014). For example, Bin et al. (2010) studied the Dinghushan plot and indicated that shrubs had weaker dispersal limitations than arbors and subarbors. Guèze et al. (2013) studied seven villages in the southwestern Amazon and indicated that the floristic patterns of large trees were explained more by environmental variables; those of small trees were explained more by geographical distances. Moreover, the large-scale dispersal ability among growth forms has been studied to a lesser degree, especially in terms of mechanism.

China, which possesses various tropical and temperate areas, provides ideal environments for investigating large-scale biodiversity patterns (Fang et al., 2012). Over the past decade, the Chinese Forest Biodiversity Monitoring Network^1^ established large stem-mapping plots located along a latitudinal gradient from temperate to subtropical and tropical forests (De Cáceres et al., 2012; Erickson et al., 2014; Song et al., 2015). Furthermore, two large stem-mapping plots were established by the Forestry research institution in Taiwan (Song et al., 2015). Here, we exploit this opportunity and analyze the turnover of woody species across temperate, subtropical, and tropical forests based on eight large stem-mapping plots.

The objectives of the current study are as follows: (1) to obtain an integrated analysis of the similarity of species composition among eight large stem-mapping plots; (2) to identify the patterns of species turnover along the latitude difference using three measures of species turnover; and (3) to assess the relative influence of environmental and neutral processes on species turnover of overall woody species and growth forms.

## Materials and Methods

### Study Sites

**FIGURE 1:**
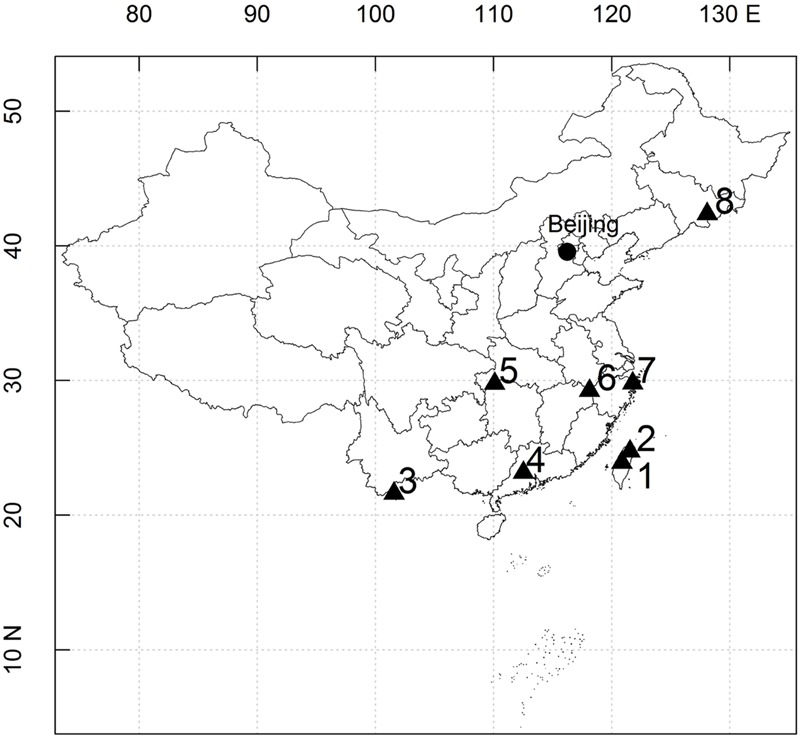
**Location of eight dynamics plots in East Asia.** (1) Lianhuachi plot; (2) Fushan plot; (3) Xishuangbanna plot; (4) Dinghushan plot; (5) Badagongshan plot; (6) Gutianshan plot; (7) Tiantongshan plot; (8) Changbaishan plot.

In this study, eight large stem-mapping plots were selected, as follows: the Lianhuachi, Fushan, Xishuangbanna, Dinghushan, Badagongshan, Gutianshan, Tiantongshan, and Changbaishan plots. Distributions of these eight plots are shown in **Figure 1**. The latitude of the plots cross ranges from 21.61 to 42.38°, and the average elevation ranges from 350.0 to 1405.5 m. The community type includes tropical rain forests, subtropical evergreen broad-leaved forests, and temperate deciduous broad-leaved forests. Census methodologies were the same for all plots: all trees with a diameter at breast height ≥1 cm were tagged and identified.

### Species and Environment Variables

In the study, we used data on the species catalog, the longitude and latitude of the plots, topographical factors [mean slope (MS, °), mean elevation (ME, m), highest elevation (HA, m), lowest elevation (LE, m)], and climate factors [mean annual precipitation (MAP, mm), relative humidity (RH, %), mean annual temperature (MAT, °C), and the mean temperature of the warmest (MTWM, °C) and coldest months (MTCM, °C)]. The species catalog was obtained from literature. Longitude and latitude, topographical factors, and climate factors were obtained from the Chinese Forest Biodiversity Monitoring Network or literature (Su et al., 2007; Hao et al., 2008; Lan et al., 2008; Lin et al., 2011; Yuan et al., 2011; Song et al., 2015). The topographical factors of the Tiantongshan plot were obtained according to the topographic map in the Biodiversity Monitoring Network; and calculation methods were based on the methods by Harms et al. (2001) and Valencia et al. (2004). Information on geography and climate for the eight study sites are shown in **Table 1**.

**Table 1:** Information on geography and climate for eight study sites.

Plot name	Plot size (ha)	Latitude (°)	Longitude (°)	Mean elevation (m)	Mean slop (°)	Mean annual temperature (°C)	Mean annual Precipitation (mm)	Relative humidity (%)
Lianhuachi plot	25	23.90	120.87	756.00	35.00	25.0	2211.0	95.0
Fushan plot	25	24.75	121.55	675.00	10.00	18.3	4237.0	95.0
Xishuangbanna plot	20	21.61	101.57	789.21	11.33	21.8	1493.0	85.0
Dinghushan plot	20	23.17	112.52	350.00	40.00	20.9	1985.0	80.3
Badagongshan plot	25	29.77	110.09	1405.50	36.00	11.5	2105.4	90.0
Gutianshan plot	24	29.25	118.12	580.60	37.00	15.3	1963.7	92.4
Tiantongshan plot	20	29.80	121.78	447.25	31.50	16.2	1374.7	82.0
Changbaishan plot	25	42.38	128.08	801.50	2.23	3.6	700.0	78.0

The eight plots differed in established time, region, and researchers. Thus, plant identification varied among plots. Thus, the species names in the plots were checked based on the Flora Reipublicae Popularis Sinicae (1959–2004) and Catalogue of Life China (2013). Concurrently, all species were divided into three types, namely, trees, small trees, and shrubs, based on the records of the Flora Reipublicae Popularis Sinicae (1959–2004).

Studies that used 20 m × 20 m subplots as basic units have shown that the species–area curves of all the forest community types in China are smooth for about 6 ha, which includes most of the species in the region (Hao et al., 2008; Lan et al., 2008; Zhu et al., 2008; Yang et al., 2011). In this study, every plot has an area greater than 20 ha. Thus, every plot can reflect the profile of the species composition in the region. Furthermore, the smallest plot area is 20 ha, and the largest is 25 ha; thus, the effect of area difference on large-scale species composition among plots is negligible.

### Measurement of Species Turnover Rate

Species turnover rate is the rate of dissimilarity among species composition across all possible plot pairs along the environmental gradient. The slope of the relationship between the species turnover and environmental divergence measures species turnover rate. Jaccard’s index (β_j_; Jaccard, 1912), Sorenson’s index (β_s_; Sorensen, 1948), and Simpson’s index (β_sim_; Lennon et al., 2001) measure turnover rate of species composition. β_j_ and β_s_ are two widely employed indices and are independent of α-diversity (Jost, 2007). β_sim_ controls for local gradients in species richness (Lennon et al., 2001; Wang et al., 2012b).

$$\begin{array}{l} \;\beta_{\text{j}}=1-c/\left(a+b+c\right)=\left(a+b\right)/\left(a+b+c\right)\\ \;\beta_{\text{s}}=1-2c/\left(a+b+2c\right)\;=\left(a+b\right)/\left(a+b+2c\right)\\ \;\beta_{\text{sim}}=1-c/\left[\min\left(a,b\right)+c\right]\;=\min\left(a,b\right)/\left[\min\left(a,b\right)+c\right] \end{array}$$

where *a* and *b* are the numbers of species only occurring in the focal and neighboring plots, respectively, and *c* is the number occurring in both.

### Data Analysis

We used detrended correspondence analysis (DCA) for the ordination of plots. DCA is an effective method in vegetation analysis. In our study, we conducted DCA using a plot-species matrix using relative abundance data to analyze the similarity of the species composition among plots.

Environmental variables: We used the climate and topographical factors to determine the environmental divergence between pairs of sites: MAP, RH, MAT, MTWM, MTCM, MS, ME, MA, and LE. All environmental variables were normalized as: *x’* = (*x* - mean(*x*)) / standard deviation (*x*), where *x* is a variable. Several studies have shown that most environmental variables are highly correlated with each other in China (Wang et al., 2012a). To avoid strong multicollinearity in regression models, we did not use original environmental variables as response variables. Instead, we conducted a principle component analysis (PCA) for all environmental variables and extracted the first six PC axes, which contained 95.19% of the total variance in the original environmental variables. In addition, longitude and latitude data of all the plots as shown in **Table 1**. Latitude difference is the difference in latitude values between the plot-pair.

Spatial variables: In our study, principal coordinates of neighbor matrices (PCNM) were used to obtain the spatial variable as a response variable based on the latitude and longitude of each plot. PCNM variables represent the spatial relationship among sites more accurately than Euclidean distance matrix and geographic coordinates (Legendre et al., 2009).

To account for effects of variation in γ-diversity, we explored the relationship between β- and γ-diversities using simple regression, with significance at *p* < 0.05. γ-Diversity is the number of species occurring in each plot-pair. Moreover, a null modeling approach was used to calculate the β-deviation according to Kraft et al. (2011). β-Deviation is currently the most widely used method (Myers et al., 2013).

To explore the influence of environment and space on β-diversity, we adopted multiple generalized linear models (GLM) using the calculated β_j_, β_s_, and β_sim_ as dependent, and aforementioned environment (MAP, RH, MAT, MTWM, MTCM, MS, ME, MA, and LE) and PCNM variables as response variables. The adjusted *R*^2^ of a model is used to represent the explanatory power of environmental and PCNM variables on species turnover. To avoid overestimation resulting from a large number of response variables, we compared the values of the Akaike information criterion (AIC) to select the best environmental model and spatial model separately. For models with the same number of variables, the model with the smallest AIC was selected. Model selection stopped when any added variable increased the AIC (Tang et al., 2012b).

Partial regression analyses were used to further compare the effects of environmental and spatial processes. In the partial regression analyses, β_j_, β_s_, and β_sim_ were dependent, and environment variables (MAP, RH, MAT, MTWM, MTCM, MS, ME, MA, and LE) and PCNM variables were the response variables. Partial regression divided the variance in species turnover index into four parts: pure spatial effects, pure environmental effects, spatially structured environmental effects, and residual variance.

All analyses were conducted in R 2.15 (R Core Development Team^2^). DCA and PCA were performed using the “vegan” package (Oksanen et al., 2007). PCNM was performed using the “pcnm” package (Blanchet et al., 2008).

## Results

### Structural Characteristics of Plant Community

**FIGURE 2:**
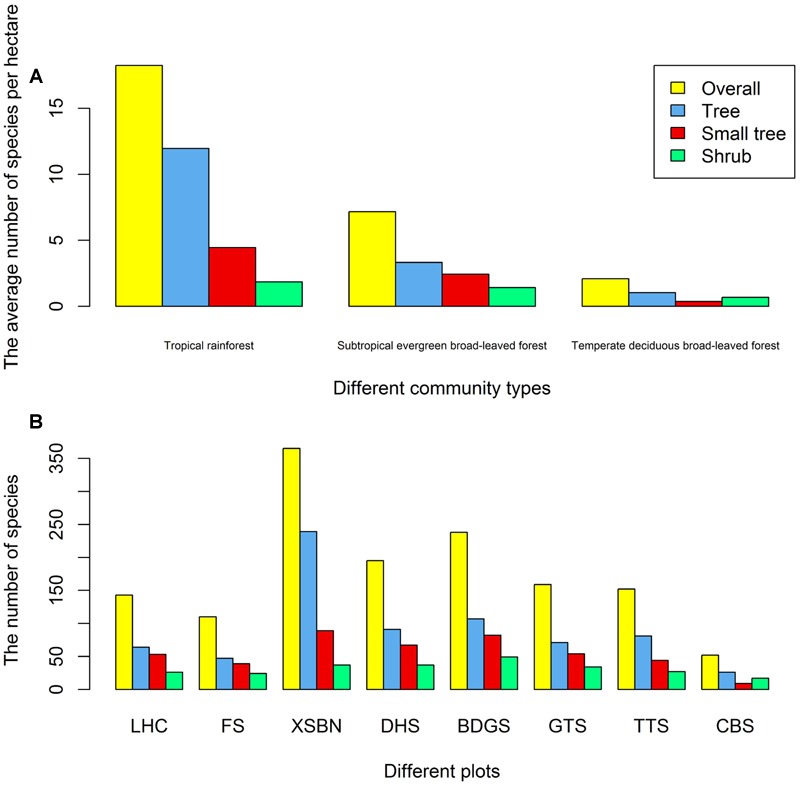
**Number of species in different community types **(A)** or different plots **(B)**.** Abbreviations: LHC, Lianhuachi plot; FS, Fushan plot; XSBN, Xishuangbanna plot; DHS, Dinghushan plot; BDGS, Badagongshan plot; GTS, Gutianshan plot; TTS, Tiantongshan plot; CBS, Changbaishan plot.

A total of 1025 woody species were included in the final dataset, encompassing 355 genera in 100 families. We classified all plots into three vegetation types based on their thermal characteristics and water availability, as follows: tropical rain forest, subtropical evergreen broad-leaved forest, and temperate deciduous broad-leaved forest (**Figure 2A**). The species diversity differed significantly among forest types. Among the eight plots, the Xishuangbanna plot belonged to the tropical rain forest; therefore, its species diversity is the highest among all plots (Overall: 365, tree: 239, small tree: 89, shrub: 37). The Changbaishan plot belonged to the temperate deciduous broad-leaved forest. Therefore, its species diversity is the lowest among all plots (Overall: 52, tree: 26, small tree: 9, shrub: 17; **Figure 2B**).

**FIGURE 3:**
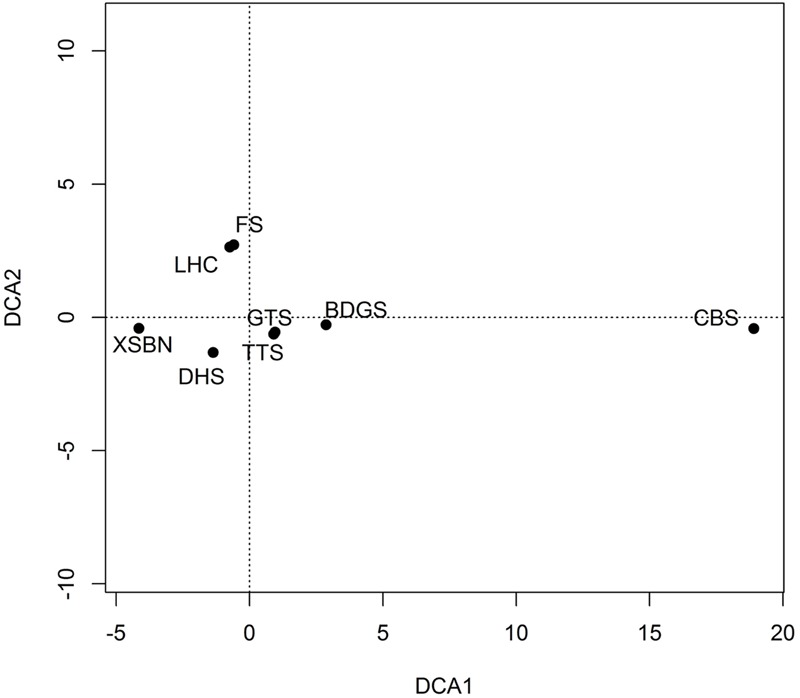
**DCA analysis of plant species composition among eight dynamics plots.** The plot codes are the same as in **Figure 2**.

The result of plot ordination is shown in **Figure 3**. The similarity between the Lianhuachi and Fushan plots is the highest in species composition. The similarities among the Dinghushan, Badagongshan, Gutianshan, and Tiantongshan plots are highest in species composition. The similarities between the Changbaishan and the other seven plots are the smallest because of far distance and large environmental differences.

### Patterns of Species Turnover

**FIGURE 4:**
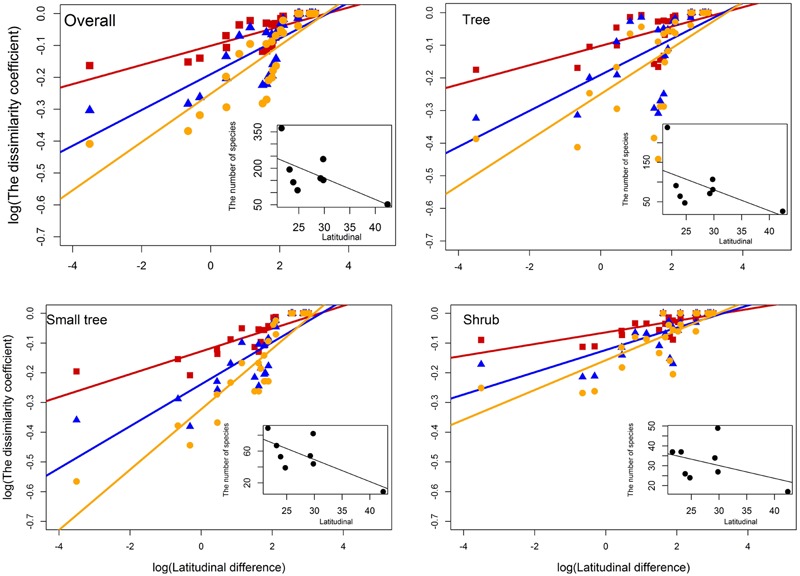
**Patterns of overall species turnover (β_j_: *R* = 0.5895^∗∗∗^; β_s_: *R* = 0.5824^∗∗∗^; β_sim_: *R* = 0.6929^∗∗∗^), trees (β_j_: *R* = 0.4009^∗∗∗^; β_s_: *R* = 0.4011^∗∗∗^; β_sim_: *R* = 0.4144^∗∗∗^), small trees (β_j_: *R* = 0.6859^∗∗∗^; β_s_: *R* = 0.6764^∗∗∗^; β_sim_: *R* = 0.8175^∗∗∗^), and shrubs (β_j_: *R* = 0.5250^∗∗∗^; β_s_: *R* = 0.5247^∗∗∗^; β_sim_: *R* = 0.6021^∗∗∗^) in different forest dynamics plots along the latitude difference.** Red square and line represent β_j_, blue triangle and line represent β_s_, and yellow dot and line represent β_sim_. The subset shows the relationship between the number of species and latitudinal across multiple forest dynamics plots. Latitude difference is the difference in latitude values between the plot-pair.

β_j_, β_s_, and β_sim_ increased significantly with increasing latitude difference of overall species, trees, small trees, and shrubs. The species turnover rates that crossed the latitudinal difference were 0.030, 0.056, and 0.076 for β_j_, β_s_, and β_sim_ of overall woody species; 0.030, 0.055, and 0.070 for β_j_, β_s_, and β_sim_ of trees; 0.038, 0.071, and 0.102 for β_j_, β_s_, and β_sim_ of small trees; and 0.020, 0.038, and 0.050 for β_j_, β_s_, and β_sim_ of shrubs, respectively (**Figure 4**). The species turnover rate of shrubs was less than that of trees and small trees along the latitudinal difference. The number of species declined significantly with increasing latitude of overall woody species, trees, small trees, and shrubs.

### Relationship between β- and γ-Diversity

**FIGURE 5:**
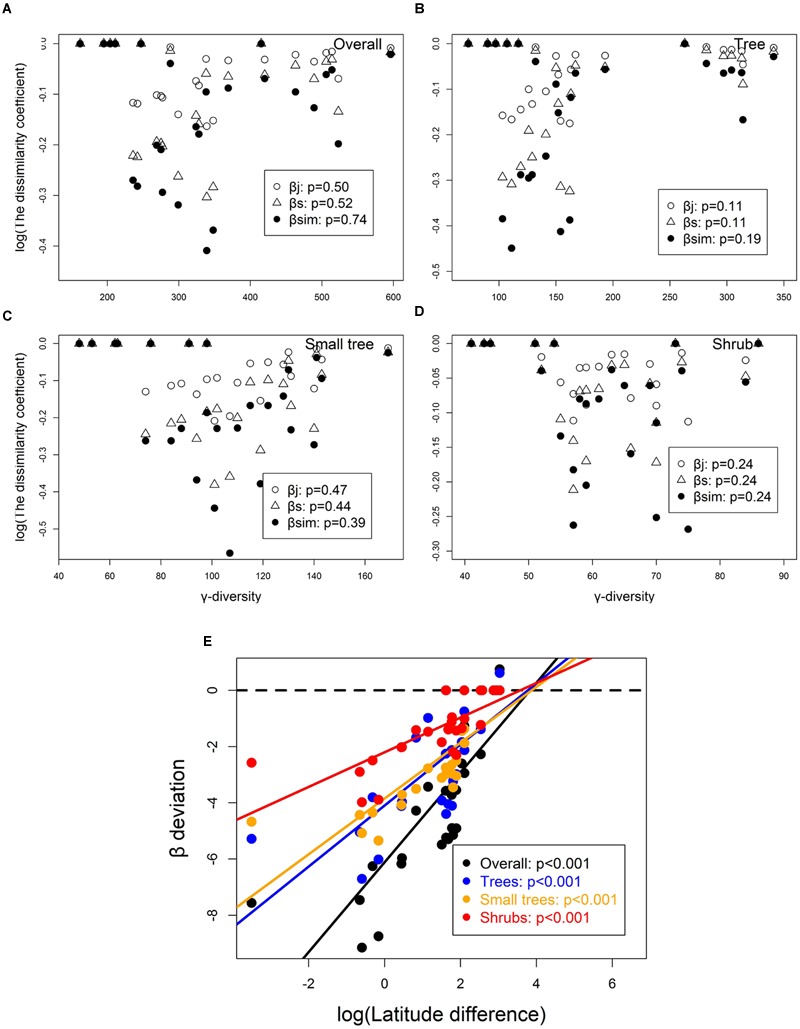
**Relationship between β- and γ-diversities for **(A)** overall, **(B)** tree, **(C)** small tree, and **(D)** shrub.** Open dots represent β_j_, gray triangles represent β_s_, and solid dots represent β_sim_. A standard effect size of β-diversity deviations from a null model that corrects for γ dependence across with latitude difference **(E)**.

γ-diversity did not significantly affect β_j_, β_s_, and β_sim_ for overall species, trees, small trees, and shrubs (**Figures 5A–D**). Moreover, β-deviation increased along latitudinal difference for overall species (*R*^2^ = 0.66), trees (*R*^2^ = 0.58), small trees (*R*^2^ = 0.67), and shrubs (*R*^2^ = 0.54; **Figure 5E**). Therefore, after correcting the differences in species pool size (γ-diversity), β-diversity had significant difference along latitudinal difference.

### Determinants of Species Turnover

The first six PC axes and four PCNM variables were used to assess the effects of the environment and space. The best environmental models and spatial models are shown in **Table 2**. For overall woody species, environment explained 36.30, 37.20, and 48.48% of the total variance in β_j_, β_s_, and β_sim_, respectively; and spatial factors explained 47.92, 48.39, and 41.38% of the total variance in β_j_, β_s_, and β_sim_, respectively.

**Table 2:** Relationships between β-diversity and PC variables or PCNM variables.

Life form	β_j_			β_s_			β_sim_		
	Factors	Cum.*R^2^* (%)	AIC	Factors	Cum.*R^2^* (%)	AIC	Factors	Cum.*R^2^* (%)	AIC
Overall	PC1	11.10	-80.616	PC1	10.90	-50.891	PC1	12.32	-42.065
	PC2	28.10	-85.255	PC2	29.60	-56.134	PC2	37.80	-50.095
	PC5	34.00	-86.624	PC5	35.00	-57.362	PC5	45.74	-52.804
	PC6	36.30	-86.753	PC6	37.20	-57.461	PC6	48.48	-53.359
	PCNM1	39.13	-90.451	PCNM1	39.96	-61.168	PCNM1	33.30	-48.176
	PCNM2	41.13	-90.479	PCNM2	42.35	-61.327	PCNM3	34.45	-49.732
	PCNM3	47.92	-92.794	PCNM3	48.39	-63.361	PCNM4	41.38	-50.795
Tree	PC1	6.32	-71.364	PC1	6.60	-42.659	PC1	5.99	-33.010
	PC2	13.61	-71.578	PC2	15.10	-44.235	PC2	20.34	-36.432
	PC5	19.77	-73.657	PC5	20.70	-45.193	PC5	29.79	-38.863
	PC6	27.83	-75.618	PC6	28.10	-46.919	PC6	36.70	-40.764
	PCNM1	39.68	-82.812	PCNM1	40.95	-54.586	PCNM1	34.28	-42.316
	PCNM2	43.77	-83.741	PCNM2	45.34	-55.698	PCNM2	38.02	-42.949
	PCNM3	50.14	-86.026	PCNM3	51.31	-57.863	PCNM3	45.93	-45.654
							PCNM4	50.70	-47.267
Small tree	PC1	11.23	-72.495	PC1	11.12	-43.853	PC1	13.80	-33.389
	PC2	42.73	-82.998	PC2	44.75	-55.320	PC2	49.05	-46.165
	PC4	44.49	-82.963	PC4	46.24	-55.187	PC5	53.47	-47.682
	PC5	49.07	-84.412	PC5	50.53	-56.557			
	PCNM1	29.86	-77.619	PCNM1	30.95	-50.415	PCNM1	27.67	-37.949
	PCNM2	30.38	-78.921	PCNM2	32.00	-49.923			
	PCNM3	36.18	-79.337	PCNM3	36.83	-50.994			
Shrub	PC1	11.09	-98.995	PC1	10.95	-67.546	PC1	13.51	-57.945
	PC2	26.46	-103.040	PC2	27.30	-71.928	PC2	28.71	-62.075
	PC3	33.55	-104.830	PC3	34.69	-73.872	PC3	32.50	-62.649
	PC5	36.84	-105.360	PC5	37.90	-74.391	PC5	35.72	-63.132
	PCNM1	17.76	-101.020	PCNM1	17.93	-69.67	PCNM1	18.49	-59.484

**FIGURE 6:**
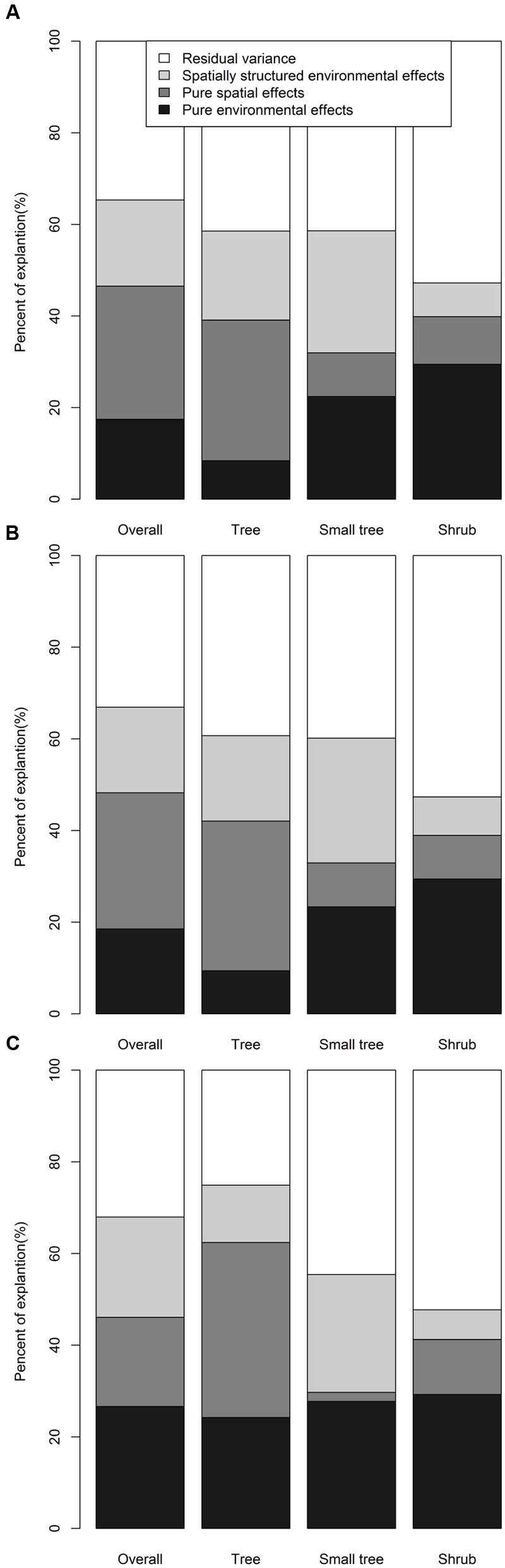
**Partial regression analyses for the effects of environmental and spatial processes on the **(A)** β_j_, **(B)** β_s_, and **(C)** β_sim_ of species composition**.

Partial regressions indicated that the effects of pure spatial and spatially structured environments on β_j_, β_s_, and β_sim_ were stronger than those of pure environmental factors for overall woody species (**Figure 6**). For trees and small trees, the effects of pure spatial and spatially structured environmental factors on β_j_, β_s_, and β_sim_ were stronger than those of pure environmental factors, whereas the effects of pure environmental factors on β_j_, β_s_, and β_sim_ were stronger than those of pure spatial and spatially structured environmental factors for shrubs. Moreover, the effects of pure environmental factors on β_j_, β_s_, and β_sim_ for trees were the lowest, and the effects of pure environmental for shrubs were the highest.

## Discussion

Large stem-mapping plots have been established in different regions worldwide (Erickson et al., 2014). Many scholars have studied large stem-mapping plots, such as functional traits (Liu et al., 2016) and species coexistence mechanisms (Aiello-Lammens et al., 2016). However, most of these studies were conducted in local areas, and integrated large-scale analyses have been poorly studied. Various ecosystems worldwide are interconnected. Thus, integrating comparative large scale analyses can reveal the rules of forest community distributions and species coexistence mechanisms. In this study, we used comparative analysis to analyze the species composition similarities of eight large forest plots. As expected, smaller distances between the plots tended to increase similarity in species composition. Moreover, species diversity decreased with increasing latitude.

Several studies have shown that species diversity generally increases with decreasing latitude (Rodríguez and Arita, 2004; Qian and Ricklefs, 2007; Kraft et al., 2011; Wang et al., 2012b). However, different methods, including the sampling scale and measures of species turnover, have been used in different studies. Sampling scale in previous studies usually were small plots (e.g., 10 m × 10 m or 20 m × 20 m), and the sampling scales in this study were 20 or 25 ha plots. Moreover, measures of species turnover in previous studies usually used one method. To avoid the error caused by using different methods of measurement, three different methods were employed to measure the species turnover rate. Based on the forest dynamics plots in this study, the patterns of species turnover increased significantly with increasing latitude differences for β_j_, β_s_ and β_sim_ of overall woody species, trees, small trees, and shrubs, respectively. These results are consistent with those obtained in previous studies (Rodríguez and Arita, 2004; Qian and Ricklefs, 2007; Kraft et al., 2011; Wang et al., 2012b). The reasons for the increase in species turnover rate with increasing latitude differences are complicated; among the reasons, latitudinal gradients in climatic tolerance and sampling effect of the species pool are the most reported (Wang et al., 2012b; Morin and Lechowicz, 2013). The hypothesis of latitudinal gradients in climatic tolerance claims that species are more climatically tolerant in high than in low latitudes. Lower climatic tolerance may further lead to narrower niche breadths in tropical than in temperate mountains, thereby decreasing the likelihood of co-occurrence of different species and increasing the species turnover rate (Wang et al., 2012b; Morin and Lechowicz, 2013).

The sampling effect hypothesis claims that variation in β-diversity across broad biogeographic gradients is more likely to be driven by γ-diversity than by differences in the mechanisms of community assembly (Kraft et al., 2011; Myers et al., 2013; Xu et al., 2015). In the present study, species pool did not significantly affect the pattern of species turnover for overall species, trees, small trees, and shrubs. These results indicated that the sampling effect hypothesis may not be applicable to the latitudinal pattern of species turnover, at least in this system. The results are inconsistent with those obtained in previous studies (Kraft et al., 2011; Myers et al., 2013; Xu et al., 2015). This inconsistency may be due to the use of latitude difference (not latitude) in our study. Latitude difference is similar to the distance between plots. The relationship between β- and γ-diversity is complicated. Some new methods have been proposed to measure and interpret β-diversity, such as multivariate pairwise distances (Bennett and Gilbert, 2016). Therefore, further analysis is needed to distinguish the effects of γ-diversity in controlling latitudinal gradients of β-diversity.

Most ecological patterns and processes in nature are scale-dependent (Bin et al., 2010; Báez et al., 2015). On a local scale, observed patterns and processes on the Changbaishan plot (Yuan et al., 2011), Lianhuachi plot (Lin et al., 2011), and Gutianshan plot (Legendre et al., 2009) showed that the diversity of forests are generally equally governed by environmental and neutral processes. On a large scale, Wang et al. (2012b) showed that spatial rather than environmental processes were the primary determinants for the latitudinal gradient in β-diversity of woody species in China. Qian et al. (2005) studied eastern Asia and eastern North America and found that environmental and neutral processes contributed equally to latitudinal β-diversity. Our results supported the hypothesis that the effect of neutral processes on species turnover of woody species in East Asia is more important than that of environmental processes. Moreover, our results are consistent with those of Wang et al. (2012b). This is likely because the study areas in our study and Wang et al. (2012b) are the same. Our study area and the obtained topographical environment characteristics have greater difference with the study of Qian et al. (2005). Therefore, our results are not consistent with those of Qian et al. (2005), but our results are not exact opposites.

The mechanisms causing patterns of species turnover may differ among trees, small trees, and shrubs (Qian, 2009; Guèze et al., 2013). This study shows that the effect of neutral processes is more important than environmental processes for trees, and that the effects of the environment for trees are minimal. However, the effects of the environment are more important than neutral processes for shrubs, and the effects of environmental factors on shrubs are greatest among trees, small trees, and shrubs. These findings may be because the dispersal abilities, such as shape, weight, number, and germination period of seeds, vary among trees, small trees, and shrubs (Qian, 2009; Farrell et al., 2012). This phenomenon is also related to shrubs being more dependent on the local environmental heterogeneity (such as aspect and humidity) and canopy species distribution than other plants (Kristiansen et al., 2012). Thus, trees are more affected by neutral processes, and shrubs are more affected by environmental processes. These results contrast with the findings obtained by Guèze et al. (2013), who determined that environmental processes explained more variation for large trees than for small trees. This discrepancy may result partly from the use of spatial extents and environmental variables in the two studies. Guèze et al. (2013) focused only on seven villages in the southwestern Amazon and only used soil factors as environmental variables. By comparison, our study covered 184 ha plots and three temperature belts in East Asia. In particular, our environmental variables included climatic and topographical factors. Thus, at a large scale, neutral processes explained more variation for turnover of tree species, and environmental processes explained more variation for turnover of shrub species.

Chang et al. (2013) studied a subtropical broad-leaved plot in Taiwan and showed that better environmental data could reverse the conclusions about community assembly processes. Furthermore, this conclusion is also supported by some studies (Jia et al., 2015). In this study, environmental variables included climate and topographical factors. Soil, as one of the important environmental factors, was not considered. However, previous studies found that soil variables influence species distribution at small spatial extents (Sarr et al., 2005). Moreover, light environmental processes and soil microorganisms are two important factors that are often ignored in community ecology studies. Therefore, future research should consider more and better environmental factors and distinguish the effects of neutral and environmental processes on a community assembly.

## Conclusion

The patterns of woody species turnover increased significantly with increasing latitude differences in East Asia. Our results support the hypothesis that the effect of neutral processes is more important than the effect of environmental processes on species turnover of woody species. However, the mechanisms underlying such patterns of species turnover may differ among trees, small trees, and shrubs. Neutral processes explained more variation for turnover of tree species. Environmental processes explained more variation for turnover of shrub species at a large scale. Therefore, trees and shrubs should receive different protection strategies in future biodiversity conservation efforts.

## Author Contributions

YY originally formulated the idea, YC and ZY developed methodology, PL, RC and HJ conducted fieldwork, YC and ZY performed statistical analyses and wrote the manuscript.

## Conflict of Interest Statement

The authors declare that the research was conducted in the absence of any commercial or financial relationships that could be construed as a potential conflict of interest.

## Footnotes

1 http://www.cfbiodiv.org/

2 http://www.Rproject.org
